# The E3 ligase Mule protects the heart against oxidative stress and mitochondrial dysfunction through Myc-dependent inactivation of Pgc-1α and Pink1

**DOI:** 10.1038/srep41490

**Published:** 2017-02-02

**Authors:** Keith Dadson, Ludger Hauck, Zhenyue Hao, Daniela Grothe, Vivek Rao, Tak W. Mak, Filio Billia

**Affiliations:** 1 Toronto General Research Institute, Toronto, 100 College St., M5G 1L7, Ontario Canada; 2 Campbell Family Cancer Research Institute, Princess Margaret Hospital, Toronto, Ontario, Canada; 3 Division of Cardiovascular Surgery, UHN, Toronto, ON, M5G 2C4, Canada; 4 Division of Cardiology, University Health Network (UHN), Toronto, Ontario, Canada; 5 Heart and Stroke Richard Lewar Centre of Excellence, University of Toronto, Toronto, Ontario, Canada; 6 Institute of Medical Science, University of Toronto, 1 King’s College Circle, Toronto, M5G 1A8, Ontario Canada

## Abstract

Cardiac homeostasis requires proper control of protein turnover. Protein degradation is principally controlled by the Ubiquitin-Proteasome System. Mule is an E3 ubiquitin ligase that regulates cellular growth, DNA repair and apoptosis to maintain normal tissue architecture. However, Mule’s function in the heart has yet to be described. In a screen, we found reduced Mule expression in left ventricular samples from end-stage heart failure patients. Consequently, we generated conditional cardiac-specific Mule knockout (*Mule *^*fl*/*fl*(*y*)^*;mcm*) mice. Mule ablation in adult *Mule *^*fl*/*fl*(*y*)^*;mcm* mice prevented myocardial c-Myc polyubiquitination, leading to c-Myc accumulation and subsequent reduced expression of Pgc-1α, Pink1, and mitochondrial complex proteins. Furthermore, these mice developed spontaneous cardiac hypertrophy, left ventricular dysfunction, and early mortality. Co-deletion of Mule and c-Myc rescued this phenotype. Our data supports an indispensable role for Mule in cardiac homeostasis through the regulation of mitochondrial function via maintenance of Pgc-1α and Pink1 expression and persistent negative regulation of c-Myc.

Cardiovascular diseases are the leading causes of morbidity and mortality worldwide^1^,^2^. As such, identification of factors regulating cardiac tissue homeostasis is of great scientific and clinical import^3^. Shortly after birth, mammalian cardiomyocytes (CM) cease to proliferate by exiting the cell cycle^4^,^5^ which renders the adult mammalian heart functionally unable to repair itself following injury^6^. Instead, CM undergo hypertrophy to compensate for the ensuing hemodynamic stress manifested as cell enlargement, myofibrillar disarray and re-expression of fetal genes^7^. This process becomes maladaptive with time leading to the development of heart failure (HF) with significant morbidity and mortality.

Structural remodeling associated with protein synthesis and degradation is an important element in the pathophysiology of the failing heart. While elevated protein synthesis is a well-established process in cardiac hypertrophy, the link between proteosome mediated protein degradation and cardiac hypertrophy is less well understood^8^,^9^. The Ubiquitin-Proteosome System (UPS) represents the major protein degradation pathway involved in the regulation of many biological signaling pathways^10^. However, while inhibition of proteosomal function has proven beneficial towards maintaining cardiac homeostasis *in vivo*^11^,^12^,^13^,^14^, this approach has suffered from poor clinical translation^15^. Currently there is a greater impetus to utilize downstream specificity of the UPS achieved through targeted ubiquitination of substrate proteins by the E3 ubiquitin ligases, of which over 600 have been found within the human and mouse genomes. Indeed, harnessing E3 ubiquitin ligase specificity to regulate key factors governing cardiac homeostasis and hypertrophy holds potential for therapeutic intervention.

The E3 ubiquitin ligase Mule is encoded by the highly conserved, X chromosome-linked Huwe1 gene^16^. Mule is ubiquitously expressed as a large (482 kDa) multi-domain member of the Hect domain family that attaches a 48-linked lysine polyubiquitin chain onto its target substrates^17^,^18^. Mechanistically, Mule functions as a tumor suppressor by targeting substrates, including Cdc6^19^, Arf^18^, Myc^20^, and p53^18^ for proteasomal degradation. Interestingly, systemic Mule deletion is embryonic lethal in mice by E12.5^18^,^21^, although by E9.5 and E10.5 the embryos display an enlarged heart and decreased body size suggesting a regulatory role for Mule in growth-related processes^21^. The role of Mule in the adult heart has not been investigated as-of-yet, and we recognized the importance of investigating the potential role of Mule as a regulator of cardiac growth^22^,^23^.

The Mule target, c-Myc (Myc), is ubiquitously expressed and encodes a nuclear phosphoprotein that functions as a highly pleiotropic transcription factor. In mitotic cells, Myc promotes proliferation, apoptosis, DNA damage, sensitivity to reactive oxygen species (ROS), glycolysis and glutamine catabolism^24^. Interestingly, ectopic overexpression of Myc in transgenic mice evokes cardiac hypertrophy without left ventricular (LV) dysfunction^25^, and controls mitochondrial (mt) energy metabolism through regulation of Pgc-1α^26^. Conversely, genetic inactivation of Myc in adult mice attenuates cardiac hypertrophy in response to pressure overload via downregulation of glycolytic factors and mt biogenesis^27^. Although Myc protein levels are increased within hours of the induction of pathological cardiac stress^26^,^28^, molecular mechanisms endogenously regulating Myc levels in the heart remain elusive.

In this study, we describe changes in Mule expression in patients with end-stage HF. In addition, we evaluate the alterations in cardiac function, energy metabolism and ROS defense in the presence or absence of Mule and/or Myc in the adult mouse heart. We are able to demonstrate that Myc is a specific Mule target and that the loss of Mule severely impacts redox homeostasis and mt function through negative regulation of Pgc-1α and Pink1, leading to oxidative stress, energy deprivation and cardiac dysfunction. Both processes are early, Myc-dependent events, and causative for the development of pathological hypertrophy, LV dysfunction, and premature mortality. Our data establishes Mule as an important regulator of cardiac homeostasis through the persistent downregulation of Myc and the upregulation of Pgc-1α and Pink1 expression.

## Materials and Methods

### Cardiac-specific *Mule* ^*fl*/*fl*(*y*)^, *Myc* ^*fl*/*fl*^ and *Mule* ^*fl*/*fl*(*y*)^ *;Myc* ^*fl*/*fl*^ *;mcm* conditional mutant mice

All animal usage and experimental methods described in this study was in accordance with approved institutional animal care guidelines of the University Health Network (Animal utilization protocol #1815 and #1379, Canadian Council in Animal Care). All animals used in this study were 10 weeks old (22–26 g) at the beginning of experimentation. All experiments used isogenic littermate controls of matched age and sex.

We generated *Mule*^*fl*/*fl*(*y*)^ as previously described^1^. *Myc*^*fl*/*fl*^ mice were generously provided by Dr. Frederick W. Alt (Department of Genetics, Harvard Medical School, Boston, MA 02115, USA). *Mule*^*f*/*f*(*y*)^ and *Myc*^*fl*/*fl*^ mice were previously backcrossed into a C57BL/6J background for 7 generations. The *mcm* strain (Tg(Myh6-cre/Esr1*)1Jmk/J) was from Jackson (Bar Harbor, ME 04609 USA). In these mice, the cardiac muscle α-myosin heavy chain 6 promoter drives the expression of Cre (c) recombinase fused to two mutant (m) estrogen-receptor ligand-binding domains (*mcm*) when exposed to Tam. We crossed *mcm* transgenic mice on a C57BL/6J background with mice carrying the conditional alleles *Mule*^*fl*/*fl*(*y*)^ and *Myc*^*fl*/*fl*^ to obtain *Mule*^*fl*/*fl*(*y*)^*;mcm* and *Myc*^*fl*/*fl*^*;mcm*. We also crossed *Mule*^*fl*/*fl*(*y*)^*;mcm* with *Myc*^*fl*/*fl*^*;mcm* mice to obtain *Mule*^*fl*/*fl*(*y*)^*;Myc*^*fl*/*fl*^*;mcm* double knockout animals referred to as *DKO* throughout this work. Because Tam and Cre expression can be toxic to cells, we included vehicle- and Tam-injected wild-type C57BL/6J, mcm and *Mule*^*fl*/*fl*(*y*)^ mice in all initial analyses of the corresponding mutants. We found that mice of these experimental groups were phenotypically indistinguishable from vehicle-injected *Mule*^*fl*/*fl*(*y*)^;mcm control animals used in this study, as judged by heart body weight ratios, ventricular fibrosis and fractional shortening (Supplementary Figure S4A–C).

After weaning, experimental male mice were housed in groups of 3–5 animals in mechanically ventilated cages (600 cm^2^; changed fortnightly) environmentally enriched by bedding and nesting materials and crawl tubes. Animals were held in a temperature-controlled environment at 19–22 °C on a diurnal 12 hour light cycle. Mice were provided free access to standard non-medicated pelleted laboratory rodent chow (Harlan) and tap water ad libitum from a portable water faucet.

DNA isolated from fresh tail snips by alkaline hydrolysis was employed for genotyping using the following primers (Invitrogen): *Mule*^*fl*/*fl*(*y*)^ forward GCCCCTGAATTGCTAGGAAGACTG; reverse CCGACCGGGTCCGAGTCCCTATT. *Myc*^*fl*/*fl*^ forward GCCCCTGAATTGCTAGGAAGACTG; *Myc*^*fl*/*fl*^ reverse CCGACCGGGTCCGAGTCCCTATT. Mcm forward AGGTGGACCTGATCATG GAG; mcm reverse ATACCGGA GATCATGCAAGC. We performed PCR analysis with Quanta Accustart Geltrac with GelDye (no. 95136–04K; VWR) and Platinum Blue Supermix (no. 12580–023; Invitrogen).

An ethanol-peanut oil emulsion of 4-Hydroxytamoxifen (Tam; H6278, Sigma-Aldrich) was prepared by completely dissolving 100 mg Tam in 5 ml highly purified ethanol (ACS reagent grade, anhydrous, absolute; no. 6590–32; Ricca Chemical, Fisher) while vortexing vigorously for 5–8 min. Peanut oil (32 ml) (P2144; Sigma-Aldrich) was added and the emulsion was again vigorously vortexed for 2 min. Then, the emulsion was sonicated on ice at highest output for 10–30 sec until it became translucent, aliquoted and stored at −20 °C for several months. Shortly before usage, Tam was melted in a 37 °C water bath, briefly vortexed, and 200–250 μl were immediately injected intraperitoneally into conscious mice. Animals were injected daily between 5–6 pm for four consecutive days (43.2 mg/kg cumulative dosage). Homologous recombination was completed 5–6 days after the last Tam-injection. Vehicle control mice were intraperitoneally injected with an ethanol-peanut oil emulsion lacking Tam.

For 5-bromo-2′-deoxyuridine (BrdU) (no. B5002; Sigma-Aldrich) labeling *in vivo*, BrdU was dissolved in PBS (10 mg/ml), aliquoted and stored at −20 °C. Conscious mice were intraperitoneally injected with 200 μl BrdU (cumulative dosage of 40 mg/kg) and sacrificed at 18 hours after the injection. The membrane permeable proteasome inhibitor MG-132 as ready made solution in DMSO was purchased from Sigma (M7449).

## Microarray Analysis

Total RNA from mouse left ventricular tissues was isolated with Trizol reagent (no. 15596026; Thermo Fisher Scientific). Phase lock Gels (no. 826754; VWR) were employed to eliminate interphase-protein contaminations. RNA quality was assessed by 260/280 and 260/230 absorption ratios employing a Nanodrop spectrophotometer (NanoDrop; Thermo Fisher Scientific), and an Agilent Bioanalyzer at the Microarray Facility, Centre for Applied Genomics, The Hospital for Sick Children (Toronto). RNA samples were processed for analysis by Affymetrix Mouse Gene 2.0 ST expression arrays at the Centre for Applied Genomics. Processing of probe level data and all subsequent analyses were performed using GeneSpring (Version 13.2; Agilent Technologies Inc.). Genome wide data from the gene expression microarrays were normalized and filtered for genes with significant expression levels (log_2_ fold change +1.3≤ or ≥−1.3; *P* < 0.05) in Tam-treated *Mule*^*fl*/*fl*(*y*)^*;mcm* compared with vehicle injected control *Mule*^*fl*/*fl*(*y*)^*;mcm (n* = 3) employing GeneSpring. Selection of differentially expressed genes in the GO gene set ‘*Cellular Metabolic Processes*’ was performed on the bases of arbitrary threshold for fold changes plus statistical significance according to the *t* test with Benjamini-Hochberg correction (log_2_ fold change ±2.0; *P* < 0.01). All microarray data were submitted to the ArrayExpress database (http://www.ebi.ac.uk/arrayexpress; accession number: E-MTAB-5112).

## Human Cardiac Samples

Human studies were conducted with the approval of the University Health Network Research Ethics Board (protocol #10–0703-TE). Informed written consent was obtained from patients with end-stage heart failure before the insertion of a left ventricular assist device or heart transplantation. Normal human adult total ventricular RNA samples were obtained from BioChain Institute, Inc. (39600 Eureka Drive, Newark, CA 94560, USA). All methods were performed in accordance with the relevant guidelines and regulations as required by the University Health Network/Toronto General Research Institute.

### Statistical analyses

Data are means ± s.e.m. as indicated at the bottom of each figure legend. We used factorial design analysis of variance (ANOVA) or τ-tests to analyze data as appropriate employing GraphPad InStat (version 3.1) and GraphPad Prism (GraphPad Software, version 7.0; La Jolla, CA 92037 USA). Significant ANOVA values were subsequently subjected to simple main effects analyses or *post hoc* comparisons of individual means using the Tukey method as appropriate. We considered *P* values of ≤ 0.05 as significant.

### Echocardiography

Echocardiography in anesthetized mice (2% isoflurane, 98% oxygen) was performed using a 15- MHz linear ultrasound transducer (Vivid7; GE). Body temperature was maintained at 37 °C. M- mode measurements of the LV end-diastolic diameter (LVEDD) and LV end-systolic diameter (LVESD) were made in triplicate from short-axis views at the level of the papillary muscle and averaged over three to six beats. LVEDD was measured at the time of the apparent maximal LV diastolic dimension, whereas LVESD was measured at the time of the most anterior systolic excursion of the posterior wall. LV fractional shortening (FS) was calculated as follows: FS = (LVEDD − LVESD)/LVEDD × 100%.

### Immunofluorescence microscopy, morphometric analyses, apoptosis and DNA synthesis assays

Mice were euthanized without anesthesia by cervical dislocation between 9–11 am. Anticoagulant was not administered. Hearts were quickly excised and rinsed in 20 ml ice-cold PBS, pH 7.4 without Ca^2+^/Mg^2+^ (no. 10010023; Thermo Fisher Scientific), fixed in 4% PBS-buffered formalin (10 ml) for 50 min at room temperature with constant agitation and incubated in 0.3 M glycine in PBS (pH 7.4; 10 ml) at 4 °C for 3–5 d. After embedding hearts in Tissue-Tek OCT Compound (Sakura, Finetek; VWR) sections were cut at 10 μm thickness using a cryostat (HM525 NX; Thermo Fisher Scientific), and mounted on histological slides (Superfrost Plus Microslides; no. 48311–703; VWR). For BrdU (5-Bromo-2′-deoxyuridine) labeling experiments, DNA was denatured in 2N HCl for 30 min post-fixation. Specimen were then permeabilized and stained with antibodies to cardiac-specific nuclear Me2f2a and BrdU. Detection of fragmented genomic DNA was performed by terminal deoxynucleotidyl transferase-mediated dUTP nick-end-labeling (TUNEL) according to the manufacturer’s instructions (no. 1684795910; Roche). These samples were co-stained with cardiac-specific anti-sarcomeric α-actinin after permeabilization. Specimen were permeabilized in 1% Triton X-100 (X100; Sigma-Aldrich) in TBS (20 mM Tris, 150 mM NaCl), pH 7.6 for 60 minutes at room temperature. Samples were incubated with primary antibodies in TBS/1% Triton X-100, for 16–20 h at room temperature without agitation. Thereafter, specimen were briefly rinsed with TBS/1% Triton X-100. For visualization, samples were incubated with secondary antibodies, diluted 100-fold in TBS/1% Triton X-100 for 1 h in the dark (Alexa Fluor 488-goat anti-rat IgG, A-11006; Alexa Fluor 555-goat anti-rabbit IgG, A-21429; Alexa Fluor 555-goat anti-mouse IgG, A-2142; Alexa Fluor 647-goat anti-mouse IgG, A-20990 (Thermo Fisher Scientific). Specimen were briefly rinsed with TBS/1% Triton X-100, and nuclear DNA was visualized with 4,6-diamidino-2-phenylindole (Dapi; 1 μg/ml in PBS) (D9542; Sigma-Aldrich). ProLong Diamond antifade reagent (no. 36965; Thermo Fisher Scientific) was applied, and samples were sealed with a coverslip. Three dimensional confocal laser scanning microscopy was performed on a Zeiss LSM700 confocal microscope and LSM Zen 2009 data acquisition software (AOMF-Advanced Optical Microscopy Facility, Ontario Cancer Institute, Toronto, ON Canada). For determination of cardiomyocyte cell size and ventricular remodeling, ventricular samples were stained with sarcomeric α-actinin and Alexa Fluor 488-conjugated wheat germ agglutinin (WGA) (W11261; Thermo Fisher Scientific). Cross-dimensions of adult cardiomyocytes and fibrotic area were determined by planimetry of immunofluorescence microphotographs using ImageJ 1.51 d (National Institutes of Health, Bethesda, MD, https://imagej.nih.gov/ij/). After recording, simple adjustments and assembly of entire and cropped microphotographs were performed employing Adobe Photoshop CS6.

### Cell fractionation, total heart tissue extracts and isolation of mitochondria

Subcellular cell fractions were prepared using the NE-PER Kit (no. 78833; Pierce) supplemented with phosphatase inhibitors (1 mM Na_3_VO4, 20 mM NaF, 10 mM β-glycerophosphate; Sigma) and with a protease inhibitor cocktail containing AEBSF, Aprotinin, Bestatin, E64, Leupeptin and Pepstatin (no. P2714; Sigma). Ventricular specimen were mechanically homogenized in RIPA Buffer (no. 9806; Cell Signaling) composed of 20 mM Tris-HCl (pH 7.5), 150 mM NaCl, 1 mM EGTA, 1% NP-40, 1% sodium deoxy cholate, 2.5 mM sodium pyrophosphate, 1 mM β- glycerophosphate, 1 mM Na_3_VO_4_, 1 mg/ml leupeptin, phosphatase and protease inhibitors. Mechanochemical assisted isolation of mitochondria from adult mouse ventricular tissues was performed using extraction buffer A (10 mM HEPES, pH 7.5; 200 mM mannitol; 70 mM sucrose; 1 mM EGTA) supplied with the Mitochondrial Isolation Kit (MITOISO1; Sigma) according to the manufacturer’s instructions. Isolated mitochondria derived from one heart were resuspended in 200 μl storage buffer (10 mM HEPES, pH 7.5, containing 250 mM sucrose, 1 mM ATP, 80 μM ADP, 5 mM sodium succinate, 2 mM K_2_HPO_4_, 1 mM DTT) (MITOISO1; Sigma), and used directly in mitochondrial complex activity assays, or aliquoted and stored at −80 °C for Western blot analysis. The protein concentration was approximately 3.0–4.0 μg/μl as determined by fluorometry (Qubit 2.0 Fluorometer; no. Q33217; Thermo Fisher Scientific).

### Mitochondrial Complex I and IV function

The relative activities of mt complex I and IV were determined employing the Complex I Enzyme Activity Assay kit (MS141; Abcam) and Complex IV Enzyme Activity Assay kit (MS444; Abcam), according to the manufacturer’s procedures except that the supplied 96-well plates were re-coated with antibodies to Complex I or IV (5.0 μg/well) and freshly purified mitochondria were used (50 μl/well). The Complex I kit determines the diaphorase-type activity of Complex I. This activity is not dependent on the presence of ubiquinone and therefore inhibitors, such as rotenone, do not inhibit Complex I activity in this assay. Importantly, the activity assay is affected by assembly deficiencies, and, thus enabled us to specifically determine the impact of decreased Ndufa4 levels (Fig. 5A) on Complex I activity. Complex IV activity was determined by following the oxidation of reduced cytochrome c. Colorimetric increases in absorbance at 450 nm for Complex I assays, and decreases in absorbance at 550 nm for Complex IV assays were recorded at room temperature for 30 min at 2 min intervals using a spectrophotometer (FlexStation 3; Molecular Devices).

### Immunoprecipitations, immunocomplex kinase assays and immunoblotting

For immunoprecipitation (IP) assays, cellular extracts in supplemented RIPA Buffer were incubated with antibodies to ubiquitin (7.5 μg) or normal rabbit IgG (7.5 μg) covalently linked to protein A agarose beads (Seize X Protein A IP Kit; no. 26149; Pierce) for 3 h at 4 °C. Immunocomplexes were washed twice with lysis buffer and boiled in SDS sample buffer (no. 7722; Cell Signaling). Protein samples were resolved by SDS-PAGE employing 4–12% and 3–8% NuPAGE pre-cast gels (Life Technologies), and PVDF membranes (iBlot; Life Technologies). The presence of ubiquitinated Mule in the immunoprecipitate was determined by immunoblotting with anti-Mule antibodies. The following secondary antibodies were employed for chemiluminescence detection of proteins: horseradish peroxidase (HRP)-conjugated anti- rabbit IgG (no. 7074; Cell Signaling), HRP-conjugated anti-mouse IgG (no. 7076; Cell Signaling), and Luminata Crescendo (WBLUR0100, Millipore). Densitometric determination of protein expression was performed with ImageJ software, version 1.49 (http://imagej.nih.gov/ij).

### ChIP assays

Potential Myc binding elements in gene promoters were identified employing the internet applications Ensembl (http://useast.ensembl.org/index.html) and TFBIND (http://tfbind.hgc.jp/). For chromatin immunoprecipitation (ChIP) analysis, ventricular tissues lysed in RIPA buffer were fixed in 1% formaldehyde for 30 min at room temperature. ChIP assays were carried out employing Myc-specific antibodies (7.5 μg) and the Agarose ChIP kit (no. 26156; Pierce) according to the manufacturer’s specifications. We used normal rabbit IgG (no. 2729; Cell Signaling) as negative control in the IP reaction. For qPCR analysis, we used 1.0 μl from a 50 μl DNA extraction and Hot-Start DNA polymerase (SensiFAST Probe No-ROX Kit; no. BIO-86020; Bioline) with 21–25 cycles of amplification.

### Detection of oxidative damage and antioxidants levels

Individual ventricular specimen were rapidly cut into 4–6 pieces, briefly rinsed in 40 ml ice-cold PBS, immediately snap frozen in dry ice-methanol, and stored at −80 °C. Assays for determination of small metabolic molecules (4-HAE, ATP, GSH/GSSG, NADPH) were deproteinized prior to analysis using the perchlorate-based precipitation based Deproteinizing Sample Preparation Kit according to the supplier’s instruction (no. K808–200; BioVision). All assays were performed according to the manufacturer’s instructions with minor modifications: 4-HAE/MDA (Bioxytech LPO-586 kit; Oxis), 8-OHdG (Bioxytech 8-OHdG-EIA kit; Oxis), ATP (ENLITEN ATP Assay System Bioluminescence no. FF200; Promega), aconitase (Bioxytech Aconitase-340 kit; Oxis), catalase (Bioxytech Catalase-520 kit; Oxis), GSH/GSSG (Bioxytech GSH/GSSG-412 kit; Oxis), NADPH (NADP/NADPH-Glo Assay; no. G9081; Promega). Each sample was measured in duplicate. Final values were normalized by the total protein concentration for each sample determined prior to precipitation.

### Determination of the Mitochondrial Membrane Potential (ΔΨm)

JC-1 (no. T3168; Thermo Fisher Scientific) exhibits potential-dependent accumulation in mitochondria, indicated by a fluorescence emission shift from green (525 nm) to red (590 nm). The depolarization ΔΨm occurs at early stages of oxidative stress and cell death. This potential-sensitive emission shift from red to green is due to concentration-dependent formation of red fluorescent JC-1 aggregates, which in turn is dependent on ΔΨm. Thus, decreases in ΔΨm are measured by decreases in the intensity of emitted red fluorescence. Isolated mitochondria (10 μl/well) in HBSS (without Ca^2+^/Mg^2+^, phenol red no. 14185–052; Thermo Fisher Scientific) were incubated with JC-1 (5 μg/ml) for 1–2 min and then treated with antimycin. JC-1 emission at 525/595 nm was recorded (2 readings/min for 30 min) using a fluorescence spectrophotometer (Flex Station 3; Molecular Devices). The rate between two time points (Δemission at 595 nm/min) was calculated in the most linear range of decline for JC-1 emission.

### Mitochondrial biogenesis and capacity

Impaired mt biogenesis is a key marker of physiologic stress or genetic mutations. Mt have multiple circular genomes (mtDNA) that are replicated independently from the nuclear genome (nDNA). The mtDNA encodes 13 core polypeptides involved in oxidative phosphorylation in addition to 22 tRNA and 12S and 16S rRNA genes for mt protein synthesis. To assess mt biogenesis, the mtDNA copy number was determined by qPCR of the mt gene cytochrome b (mtCytb) normalized to levels of a nuclear (n) encoded single-copy gene, β-actin. The ratio of mtDNA to nDNA in ventricle-injected hearts was arbitrarily set to 1. We also used mt gene transcription as a surrogate for mt capacity. The mRNA expression levels of the mtCytb gene were measured by RT-qPCR and corrected for the transcript expression of nuclear β-actin. The ratio of transcript levels of mtCytb to N-actin in ventricle-injected hearts was set to 1.

### Reverse transcription and quantitative real time PCR assays

We carried out two-step reverse transcriptase (RT) and quantitative real-time polymerase chain reactions (qPCR) on a LightCycler 480 (Roche; TMDT Core Facility) for mRNA analysis. Total RNA from mouse and human ventricular cardiac specimen was isolated with Trizol reagent (no. 15596026; Thermo Fisher Scientific), and Phase lock Gels (no. 826754; VWR) were employed to eliminate interphase-protein contaminations. We used 1.0 μg total RNA in a 20 μl reaction for first-strand cDNA synthesis employing the SensiFast cDNA synthesis kit (BIO-65053; Bioline). For qPCR, we employed 4.0 μl first-strand synthesis product, diluted 5-fold with MilliQ-grade water and the Quanta Accustart II PCR Supermix (no, 95136–04; VWR) with EvaGreen dye (no. 31000; VWR). Mule transcript levels were determined in human samples with Taqman assays employing standard human gene probes for B2m (no. HS99999907_m1) and Mule (Hs00948075_m1; Thermo Fisher Scientific). Relative quantification of Mule transcript levels was performed using the ΔΔCt method with normalization to beta-2 microglobulin (B2m) employing the data analysis module.

## Results

### Mule deficient hearts become hypertrophic and show enhanced Myc stability

**Figure 1:**
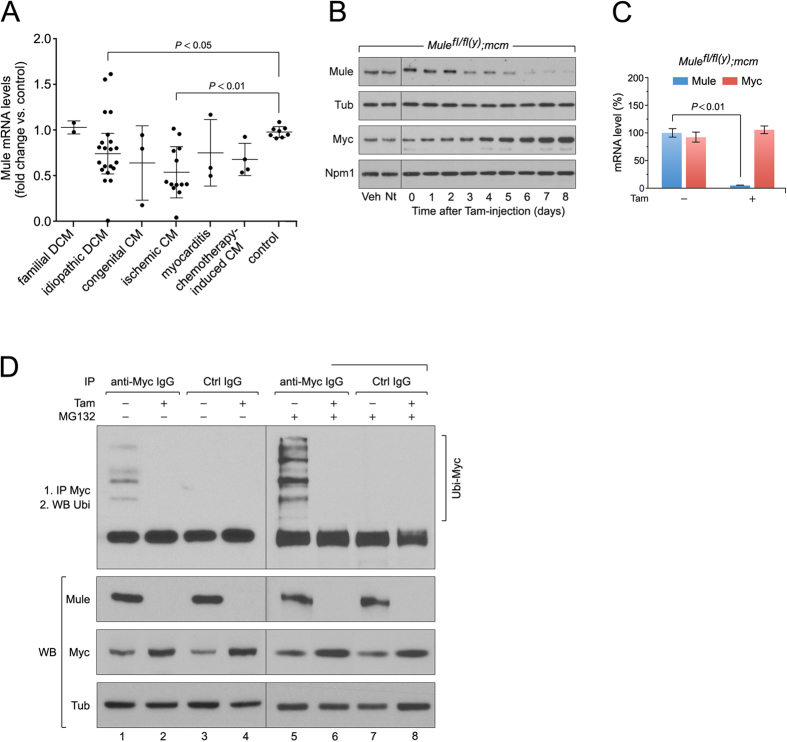
Acute genetic deletion of Mule induces concentric hypertrophy with cardiac dysfunction and premature death. (**A**) Down-regulation of Mule mRNA levels in human end-stage heart failure determined by RT- qPCR. (**B**) To specifically inactivate Mule and Myc in the adult heart, we employed a tamoxifen (Tam) inducible Cre-*loxP* system in which Cre recombinase expression is controlled by the cardiomyocyte-specific myosin heavy chain 6 promoter (*mcm*). 4-hydroxytamoxifen (Tam) was injected intraperitoneally daily for four consecutive days in 10 week-old mice. The day of the last Tam injection was set as day zero. Veh, vehicle. Nt, no treatment. Immunoblot analysis of cytoplasmic Mule and nuclear Myc levels in left ventricular extracts (60 μg total protein/lane) of *Mule*^*fl*/*fl*(*y*)^*;mcm* mice at 7 days post-Tam employing specific antibodies as indicated on the left. Animals were 12 weeks old at the time of analysis. For normalization, Western blots were probed with anti-tubulin (Tub) for cytoplasmic fractions, and anti-nucleophosmin (Npm1) for nuclear fractions. Immunoblots were repeated at least once with similar results. Transcript levels of Mule and Myc in *Mule*^*fl*/*fl*(*y*)^*;mcm* mice at 7 d post-Tam as analyzed by RT-qPCR. *n* = 4. (**C**) Mule is indispensable for Myc protein stability in the heart by regulation of its ubiquitin-mediated proteasomal degradation. At 7 d post-Tam. *Mule*^*fl*/*fl*(*y*)^*;mcm*, mice were intraperitoneally injected with the proteasomal inhibitor MG132 or vehicle for 6 hours. LV lysates were immunoprecipitated (IP) with anti-Myc antibodies or normal rabbit IgG. Ubiquitinated Myc proteins in the immunoprecipitates were identified by immunoblotting with antibodies to ubiquitin. IgG, immunoglobulin G. IP, immunoprecipitation. Ubi, ubiquitin. WB, Western blot. One representative result of three independent experiments is depicted. Data are means ± s.e.m.

We analyzed mRNA isolated from LV samples of patients with end-stage HF of varying etiologies (Supplementary Table S4). When compared to mRNA isolated from healthy hearts, Mule gene expression was significantly decreased in samples from patients with idiopathic dilated cardiomyopathy (DCM) and ischemic CM (Fig. 1A), suggesting a correlation between Mule expression and end-stage HF of these etiologies. Hence, we determined if ablation of Mule expression could induce cardiac hypertrophy and consequently HF, *in vivo*. To achieve this we crossed transgenic mice expressing Cre recombinase flanked by mutated estrogen receptors (MerCreMer; *mcm*)^29^,^30^ with mice carrying loxP flanked alleles of Mule (Mule^*fl*/*fl*(*y*)^) to obtain male *Mule*^*fl*/*fl*(*y*)^*;mcm* animals. In the absence of tamoxifen (Tam), these animals developed normally without gross cardiac abnormalities. Four consecutive daily intraperitoneal Tam injections were sufficient to deplete Mule protein levels in the heart with high recombination efficiency within 7 days (Fig. 1B). This was corroborated by decreased immunostaining for Mule protein in fixed myocardial cryosections and decreased Mule mRNA expression, assessed by RT-qPCR, in *Mule*^*fl*/*fl*(*y*)^*;mcm* mice post-Tam (Supplementary Figure S1A; Fig. 1C).

Myc is maintained at low levels in the normal heart but is robustly activated in adult CMs where it induces hypertrophy in response to stress^25^,^26^,^27^,^28^. Interestingly, we noted a significant increase in Myc protein levels in cardiac homogenates from *Mule*^*fl*/*fl*(*y*)^*;mcm* mice as early as 7 d post-Tam (Fig. 1B). Since Myc mRNA levels remained unaltered (Fig. 1C), we investigated whether Mule regulated Myc protein stability in adult CM^20^. Accordingly, endogenous Myc was immunoprecipitated from LV lysates of *Mule*^*fl*/*fl*(*y*)^*;mcm* mice injected with the proteasome inhibitor MG132 or vehicle. Ubiquitinated Myc was present in LV lysates derived from vehicle-injected *Mule*^*fl*/*fl*(*y*)^*;mcm* while ubiquitinated Myc protein was not detected in *Mule*^*fl*/*fl*(*y*)^*;mcm* lysates post-Tam (Fig. 1D). Our results suggest that Mule ubiquitinates Myc in adult CMs, contributing to its instability.

### *Mule* ^*fl*/*fl*(*y*)^ *;mcm* mice develop pathological cardiac hypertrophy and heart failure

**Figure 2:**
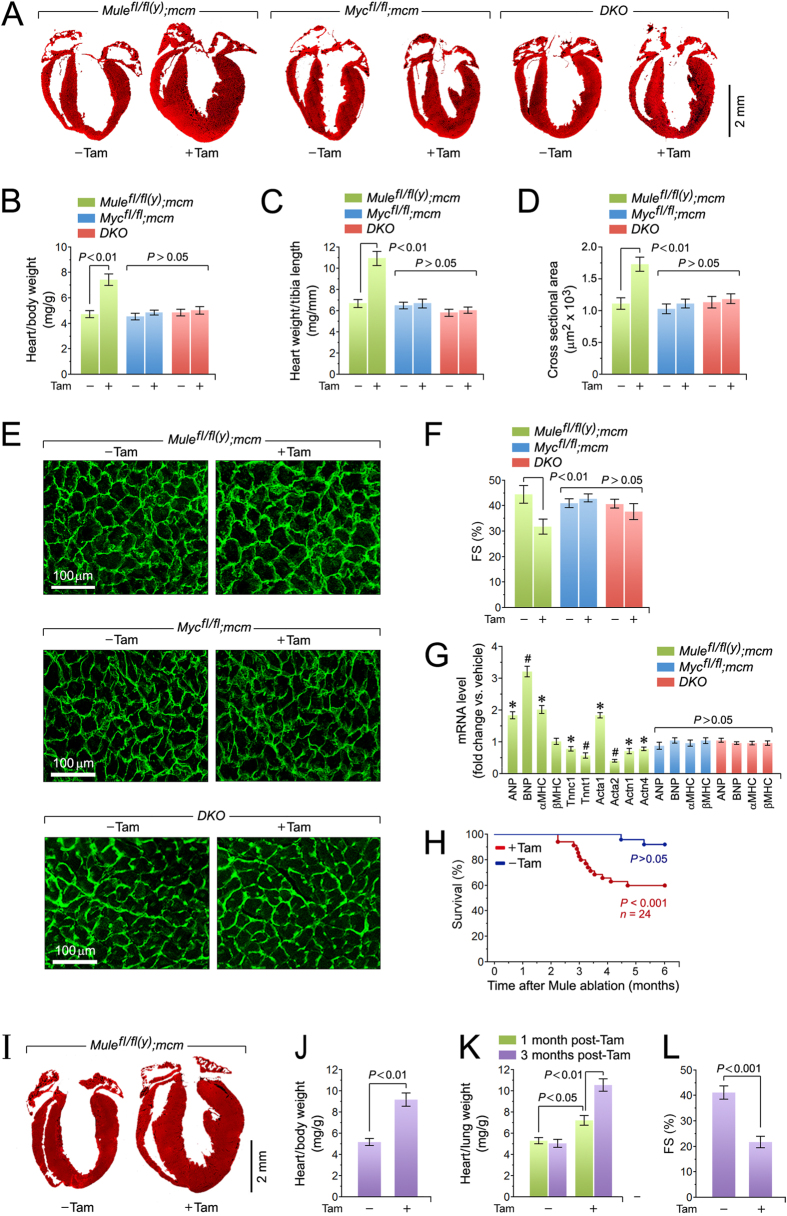
Genetic co-ablation of Myc and Mule prevents the cardiomyopathy associated with Mule-deficiency. (**A**) Representative masson staining of longitudinal cardiac sections of the indicated mice at 4 weeks post-Tam. (**B**) Heart-weight corrected for body weight of the indicated strains at 4 weeks post-Tam. Animals were 15 weeks old at the time of analysis. *n* = 28. (**C**) Heart-weight corrected for tibia length at 4 weeks post-Tam. *n* = 28. (**D**) Quantification of cross-sectional area of adult cardiomyocytes at 4 weeks post-Tam. *n* = 12. (**E**) Immunofluorescence microscopy of wheat germ agglutinin (WGA; green) stained formalin-fixed LV sections at 4 weeks post-Tam. (**F**) Fractional shortening (FS) determined by M-mode echocardiography at 4 weeks post-Tam. *n* = 4. (**G**) Expression levels of hypertrophic and sarcomeric marker genes atrial natriuretic factor (ANP), brain natriuretic factor (BNP), α-myosin heavy chain (β-MHC), β-myosin heavy chain (β-MHC), troponin C (Tnnc1), troponin T (Tnnt1), α-actin (Acta1/2) and α-actinin (Actn1/4) as analyzed by RT-qPCR at 4 weeks post-Tam. *n* = 4. *P < 0.05 versus −Tam. ^#^*P* < 0.01 versus −Tam. (**H**) Acute genetic ablation of Mule evokes premature death. Kaplan-Meier survival curves of conditional *Mule*^*fl*/*fl*(*y*)^*;mcm* mice. (**I**) Representative masson staining of longitudinal cardiac sections from *Mule*^*fl*/*fl*(*y*)^*;mcm* mice at 3 months post-Tam. (**J**) Heart-weight corrected for body weight of *Mule*^*fl*/*fl*(*y*)^*;mcm* mice at 3 months post-Tam. *n* = 14. (**K**) Lung-weight corrected for tibia length of *Mule*^*fl*/*fl*(*y*)^*;mcm* mice at the indicated time points post-Tam. *n* = 14. (**L**) Fractional shortening (FS) determined by M-mode echocardiography of *Mule*^*fl*/*fl*(*y*)^*;mcm* mice at 3 months post-Tam. *n* = 4. Data are means ± s.e.m.

To determine the physiological consequence of Mule ablation in the heart, we assessed cardiac morphology and function in *Mule*^*fl*/*fl*(*y*)^*;mcm* mice 4 weeks post-Tam. We observed cardiac hypertrophy in *Mule*^*fl*/*fl*(*y*)^*;mcm* mice post-Tam by Masson staining of longitudinal LV sections that revealed significant wall thickening in *Mule*^*fl*/*fl*(*y*)^*;mcm* hearts post-Tam (Fig. 2A). This was corroborated by significantly increased heart weight/body weight and heart weight/tibia length ratios compared to vehicle-injected *Mule*^*fl*/*fl*(*y*)^*;mcm* mice (Fig. 2B,C). Similarly, CM cross-sectional area was significantly increased in *Mule*^*fl*/*fl*(*y*)^*;mcm* post-Tam compared to vehicle-treated controls (Fig. 2D,E). Assessment of cardiac function by echocardiography revealed significantly impaired fractional shortening (32 ± 2.1%; *P* < 0.01) in *Mule*^*fl*/*fl*(*y*)^*;mcm* post-Tam (Fig. 2F). Quantitative PCR analysis of canonical hypertrophic indicators revealed increased ANP, BNP, αMHC, and Acta1 mRNA in *Mule*^*fl*/*fl*(*y*)^*;mcm* post-Tam vs vehicle control (Fig. 2G). In contrast, several sarcomeric genes were down-regulated (Tnnc1, Tnnt1, Actn1, Actn4). This was associated with premature mortality of 40% (*P* < 0.001), as early as 2.5 months post-Tam in *Mule*^*fl*/*fl*(*y*)^*;mcm* (Fig. 2H). Collectively, the findings demonstrate that Mule-deficiency induces pathological hypertrophy with decreased LV function, and premature mortality.

### Co-deletion of Mule and Myc abrogates the development of spontaneous cardiac hypertrophy and dysfunction

To assess whether development of spontaneous hypertrophy and LV dysfunction observed in the *Mule*^*fl*/*fl*(*y*)^*;mcm* mice post-Tam may be attributed to increased Myc protein expression, we generated Myc^*fl*/*fl*^;*mcm* mice. *Mule*^*fl*/*fl*(*y*)^*;mcm* and *Myc*^*fl*/*fl*^*;mcm* mice were then crossbred to generate cardiac-specific *Mule*^*fl*/*fl*(*y*)^*;Myc*^*fl*/*fl*^*;mcm* animals (designated as double knockout; *DKO*) lacking both Mule and Myc protein upon Tam injection. Immunoblot analysis confirmed Tam-dependent ablation of Myc in *Myc*^*fl*/*fl*^*;mcm* mice, and of Mule and Myc in the *DKO* mice 6 days following four daily intraperitoneal Tam injection (Supplementary Figure S2A,B). Depletion of Myc protein was further verified through immunostaining in fixed myocardial cryosections (Supplementary Figure S1B).

Assessment of LV longitudinal sections 4 weeks post-Tam injection showed no gross morphological difference between *Myc*^*fl*/*fl*^*;mcm* or *DKO* mice, in the presence or absence of Tam (Fig. 2A). Similarly, there was no evidence of cardiac hypertrophic growth in these animals (Fig. 2B–E), suggesting that ablation of Myc and co-deletion of Mule/Myc do not induce the pathological phenotype observed in Mule-deficient hearts. Neither FS (Fig. 2F) nor hypertrophic markers were significantly altered in *Myc*^*fl*/*fl*^*;mcm* or *DKO* mice (Fig. 2G).

Next, we analyzed heart function in *Mule*^*fl*/*fl*(*y*)^*;mcm* mice at 3 months post-Tam when significant morality was noted (Fig. 2H). In these animals, ventricular dilation was significantly more pronounced than in *Mule*^*fl*/*fl*(*y*)^*;mcm* mice at 4 weeks post-Tam (Fig. 2I,K). Importantly, lung weight/body weight (LBW) ratios, an index of pulmonary congestion and LV dysfunction, was markedly elevated in *Mule*^*fl*/*fl*(*y*)^*;mcm* mice at 3 months post-Tam versus controls. Notably, LBW ratios from *Mule*^*fl*/*fl*(*y*)^*;mcm* mice at 4 weeks post-Tam also showed a statistically significant increase versus vehicle controls. Examination of cardiac performance by echocardiography on viable *Mule*^*fl*/*fl*(*y*)^*;mcm* mice at 3 months post-Tam revealed severely impaired contractility, as indicated by significant decreases in fractional shortening, in comparison to vehicle-treated animals (Fig. 2L). Collectively, these findings indicate that Mule is needed to prevent the development of end-stage congestive heart failure with early death.

### Myc co-deletion protects against fibrosis and apoptosis induced by Mule deficiency alone

**Figure 3:**
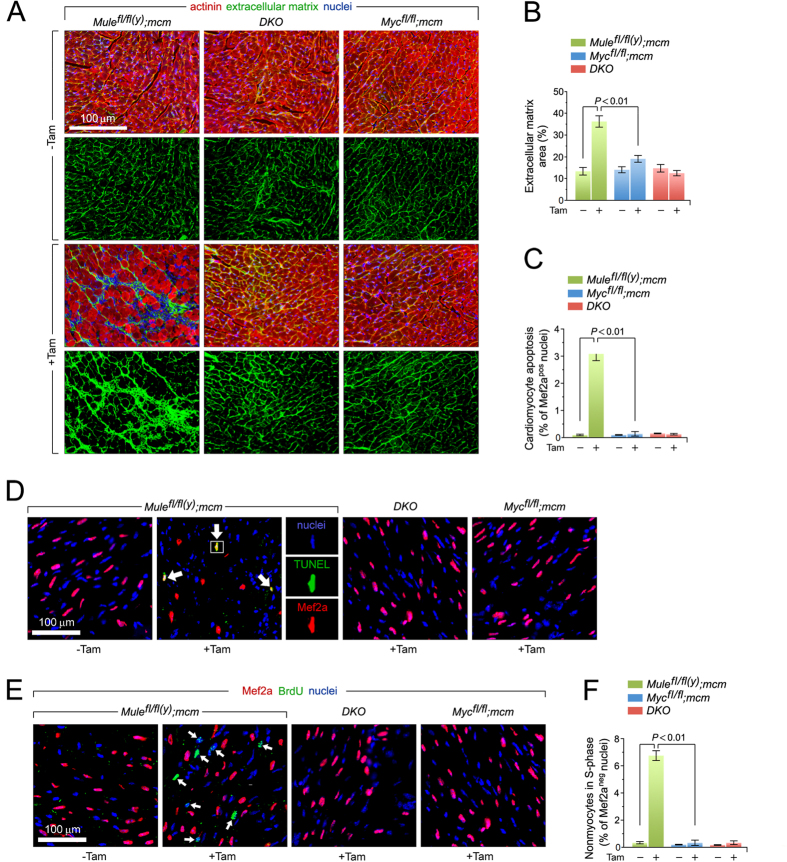
Mule-deficiency induces cardiomyocyte apoptosis and structural remodeling of the ventricular wall. (**A**) Analysis of cardiac fibrosis by immunofluorescence microscopy employing wheat germ agglutinin (WGA) staining (green) of collagen deposition in the extracellular matrix, cardiomyocyte-specific anti-actinin (red), and Dapi (blue) to visualize nuclear DNA. (**B**) Quantification of extracellular matrix area indicative of LV fibrosis shown in (**B**). *n* = 4. (**C**) Acute genetic ablation of Mule triggers cardiomyocyte apoptosis which is abrogated by co- deletion of Mule and Myc in *DKO* mice. *n* = 4. (**D**) Analysis of apoptosis in LV cardiomyocytes (white arrows) by immunofluorescence microscopy and TUNEL assays. Hearts were harvested at 8 d post-Tam. Mice were 12 weeks old at the time of analysis. Red, cardiomyocyte-specific nuclear marker, anti-Mef2a. Green, TUNEL. Blue, DAPI stain of nuclear genomic DNA. TUNEL, terminal deoxynucleotidyl transferase- mediated dUTP nick-end-labeling. (**E**) BrdU, an indicator for DNA synthesis was injected intraperitoneally at 7 d post-Tam. Animals were sacrificed 18 hours later. Quantitative analysis of cardiomyocytes in S phase was performed by immunofluorescence microscopy of LV cardiac sections employing anti-BrdU (green) and anti-Mef2a (red) antibodies. White arrows denote BrdU-positive and Mef2a-negative non-cardiomyocytes. Blue, nuclei). BrdU, 5-Bromo-2′-deoxyuridine. (**F**) Genetic ablation of Mule fails to induce cell cycle entry and DNA synthesis in adult cardiomyocytes. n = 4. Data are means ± s.e.m.

Quantitative analysis of ECM deposition revealed significant interstitial ECM accumulation in Tam-treated *Mule*^*fl*/*fl*(*y*)^*;mcm* mice (Fig. 3A,B), with patchy interstitial fibrosis observed with confocal microscopy (Fig. 3A). This was absent in *Myc*^*fl*/*fl*^*;mcm* and *DKO* (Fig. 3A,B). Apoptosis was assessed in fixed LV cryosections probed using the TUNEL assay. Numbers of TUNEL-positive nuclei were significantly increased only in *Mule*^*fl*/*fl*(*y*)^*;mcm* mice post-Tam (2.1 ± 0.3%; *P* < 0.01), but not *DKO* or *Myc*^*fl*/*fl*^*;mcm* animals (Fig. 3C,D).

Myc target genes are known regulators of both cell growth and the cell cycle^31^. Therefore, we analyzed fixed LV cryosections for evidence of CMs in S phase of the cell cycle. Confocal immunofluorescence imaging of LV sections isolated from BrdU-injected *Mule*^*fl*/*fl*(*y*)^*;mcm* mice treated with Tam revealed significant DNA synthesis of non-CM cell populations (Fig. 3E,F). There was no evidence of CMs in S phase in *Mule*^*fl*/*fl*(*y*)^*;mcm, Myc*^*fl*/*fl*^*;mc*m, or *DKO* mice with or without Tam injection.

### Genome-wide expression profiling of Mule-regulated transcripts

To understand Mule-deficiency induced hypertrophy at the transcriptome level, genome-wide RNA expression profiling was performed on LV lysates from *Mule*^*fl*/*fl*(*y*)^*;mcm* mice. Mule ablation induced a broad derangement of the cardiac transcriptome 4-weeks post Tam injection: 3,479 of 33,551 genes were differentially regulated in the *Mule*^*fl*/*fl*(*y*)^*;mcm* mice post-Tam (false discovery rate <0.5% vs untreated *Mule*^*fl*/*fl*(*y*)^*;mcm* mice) (Supplementary Figure S3A). The significant downregulation of Myc target genes (Supplementary Figure S3B), suggests an association between increased Myc levels and Mule-deficiency^32^.

**Figure 4:**
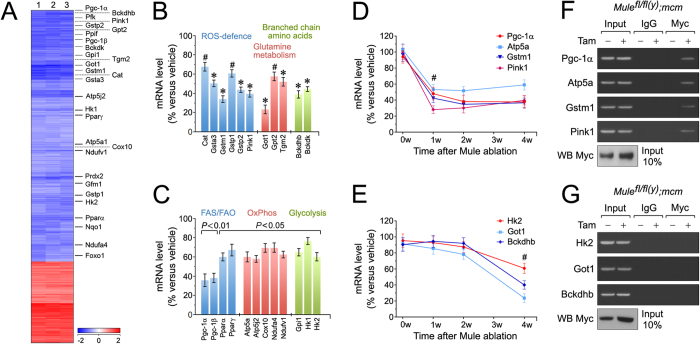
Mule inhibits Myc-dependent deterioration of cardiac function by downregulation of Pgc-1α and Pink1. Microarray-based genome-wide transcriptional profiling of LV tissue samples derived from *Mule*^*fl*/*fl*(*y*)^*;mcm* mice at 4 weeks post-Tam. Animals were 15 weeks old at the time of analysis. Depicted are differentially expressed genes that were significantly enriched for a cellular metabolic gene signature in the Mule-deficient mice (column) post-Tam relative to vehicle- injected controls. Heatmap values (log_2_ fold expression) of this metabolic gene set are shown by color and intensity of shading. Important factors of cardiac energy metabolism and detoxification of oxygen radicals are indicated on the right. Blue, repressed. Red, induced. *n* = 3 biological replicates. *P* < 0.01. Fold change >2.0. (**A**) Expression levels of key genes selected from (**A**), involved in the regulation of branched chain amino acid metabolism, glutamine metabolism and ROS defence as analyzed by RT-qPCR at 4 weeks post-Tam. *n* = 4. *P < 0.05 versus −Tam. ^#^*P* < 0.01 versus vehicle. (**B**) Expression levels of differentially enriched genes from (**A**), involved in the regulation of glycolysis, mitochondrial oxidative phosphorylation (OxPhos), fatty acid synthesis (FAS) and fatty acid oxidation (FAO) as analyzed by RT-qPCR at 4 weeks post-Tam. *n* = 4. (**D**,**E**) Time course of transcript levels of selected factors (C) participating in the regulation of important biological processes in *Mule*^*fl*/*fl*(*y*)^*;mcm* mice was analyzed by RT-qPCR. *n* = 4. ^#^*P* < 0.01 versus vehicle. (**F**) Endogenous Myc interacts with gene promoter sequences of Pgc-1α, Atp5a, Gstm1 and Pink1 *in situ*. One representative result of three independent experiments is shown. (**G**) Myc does not bind to the promoters of Hk2, Got1 and Bckdhb. LV tissue obtained from *Mule*^*fl*/*fl*(*y*)^*;mcm* mice at 7 d post-Tam was analyzed by ChIP employing anti-Myc antibodies and PCR primers specific to the selected gene promoters. Normal rabbit IgG were used for control immunoprecipitations. Input lanes of chromatin levels used for the immunoprecipitation step demonstrate equal loading. One representative result of three independent experiments is shown. Data are means ± s.e.m.

Gene Ontology (GO) analysis of the *Mule*^*fl*/*fl*(*y*)^*;mcm* transcriptome identified ‘*Cellular Metabolic Processes*’ as an enriched GO term. The majority of factors (67%; 58/87) in this gene set were downregulated. Intriguingly these genes regulate important biological processes including ROS defense (Cat)^33^, glutamine metabolism (Got1)^34^, and branched-chain amino acids (Bckdk)^35^ or were highly relevant to the regulation of myocardial FAS and FAO (Pgc-1α, mt OxPhos (Cox10) and glycolysis (Hk2) (Fig. 4A). Downregulation of these factors was confirmed by RT-qPCR (Fig. 4B,C).

To identify processes responsible for the development of the phenotype in Mule-deficient hearts, we analyzed mRNA kinetics of key regulators by RT-qPCR. Pgc-1α, Atp5a, Gstm1 and Pink1 expression declined 1 week post-Tam, and were deemed ‘early responding genes’ (Fig. 4D). Promoter binding analysis using chromatin-immunoprecipitation (ChIP) on *Mule*^*fl*/*fl*(*y*)^*;mcm* cardiac homogenates identified enhanced binding of Myc to the promoter region of the early responding genes (Fig. 4F), but not to the promoters of Hk2, Got1 and Bckdhb (Fig. 4G) whose expression was stable until 4 weeks post-Tam (Fig. 4E).

### Myc impairs oxidative phosphorylation by selective downregulation of Pgc-1α

**Figure 5:**
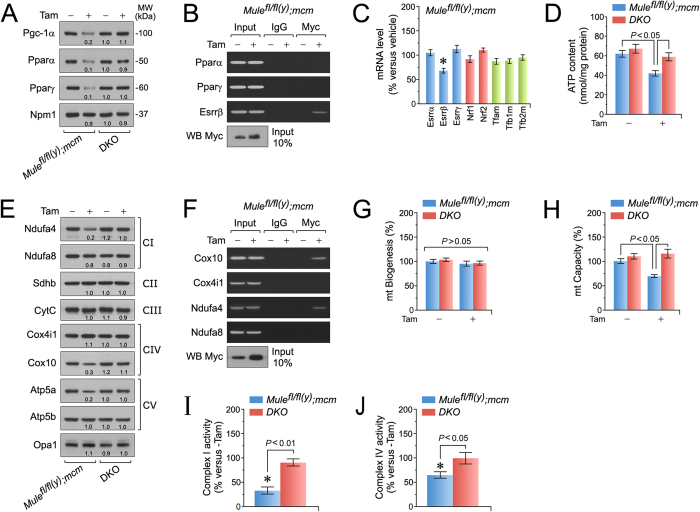
Mule is indispensable for the maintenance of mitochondrial biogenesis and bioenergetics. (**A**) Immunoblot analysis of master transcription factors regulating FAS and FAO in left ventricular extracts of *Mule*^*fl*/*fl*(*y*)^*;mcm* and *DKO* mice at 7 days post-Tam. Numbers below the individual blots indicate fold-changes in protein expression. (**B**) Endogenous Myc interacts with gene promoter sequences of Esrrβ, but is not associated with the promoters of Pparα and Pparγ as analyzed by ChIP. Esrrβ, estrogen related receptor, beta. (**C**) Transcript expression of key factors regulating FAS/FAO, redox homeostasis and mt biogenesis in left ventricular samples of *Mule*^*fl*/*fl*(*y*)^*;mcm* mice was determined by RT-qPCR at 4 weeks post-Tam. *n* = 4. *P < 0.05 versus vehicle. (**D**) ATP levels in left ventricular tissue extracts from *Mule*^*fl*/*fl*(*y*)^*;mcm* mice at 7 d post-Tam. *n* = 4. (**E**) Immunoblot analysis of important constituents of the mt electron transport chain participating in oxidative phosphorylation in LV extracts of *Mule*^*fl*/*fl*(*y*)^*;mcm* versus *DKO* mice at 7 days post-Tam. Equal loading of bands was confirmed by reprobing membranes with mt- specific anti-Opa1 antibodies. (**F**) Myc interacts with gene promoter sequences of OxPhos components of Ndufa4 (CI, complex 1) and Cox10 (CIV, complex 4) as analyzed by ChIP from isolated LV genomic DNA from *Mule*^*fl*/*fl*(*y*)^*;mcm* mice at 7 d post-Tam. (**G**) Mt biogenesis, defined as relative DNA copy number of mt encoded Cytb gene normalized to the copy number of the nuclear gene Npm1, was determined by qPCR of isolated LV mt from *Mule*^*fl*/*fl*(*y*)^*;mcm* and *DKO* mice at 7 d post-Tam. *n* = 4. (**H**) Mt capacity, defined as relative mRNA levels of the nuclear gene Ndufa8, a subunit of OxPhos complex I, normalized to Npm1 transcript expression, was determined by RT-qPCR of total RNA isolated from left ventricular tissue of the indicated wild type (−Tam) and mutant strains (+Tam). *n* = 4. (**I**) Activity of OxPhos complex I in *Mule*^*fl*/*fl*(*y*)^*;mcm* and *DKO* mice at 7 d post-Tam. *n* = 4. *P < 0.01 versus vehicle. (**J**) Activity of OxPhos complex IV in *Mule*^*fl*/*fl*(*y*)^*;mcm* mice or *DKO* animals at 7 d post-Tam. *n* = 4. *P < 0.05 versus vehicle. Data are means ± s.e.m.

To determine whether activated Myc decreases major regulators of mt function and FAO, we performed immunoblot and RT-qPCR analyses of LV lysates. Notably protein levels of Pgc- 1α and Pparα/γ were markedly lower in *Mule*^*fl*/*fl*(*y*)^*;mcm* mice post-Tam (Fig. 5A). However, Myc did not bind the Pparα/γ promoters (Fig. 5B), suggesting that Pgc-1α is regulated by Myc whereas Pparα/γ is not. Interestingly, the promoter region of Esrrβ did immunoprecipitate with Myc (Fig. 5B) and this correlated with the decrease in Esrrβ transcript expression in *Mule*^*fl*/*fl*(*y*)^*;mcm* mice post-Tam (Fig. 5C). We also observed that expression of the mt transcription factors Esrrα/γ, Nrf1/2, and Tfam/Tfbm, remained unchanged in the presence of activated Myc (Fig. 5C) suggesting that Myc-mediated regulation of mt proteins is achieved through binding of nuclear factors^36^ including Essrβ in the heart.

It has been well established that impaired ATP production is detrimental for contractility^37^,^38^. Consistent with this, significant decreases in ATP levels were restricted to Mule-deficient hearts compared to vehicle controls (Fig. 5D). Next we determined whether reduced ATP levels were related to Myc-mediated repression of mt complex proteins. As shown in Fig. 5E, Mule ablation induced a marked decrease in Ndufa4, Cox10, and ATP5a expression levels, all crucial components of respiratory chain complex I, IV and V. This effect was abrogated in *DKO* mice post-Tam (Fig. 5E). Moreover, ChIP analysis demonstrated increased Myc binding to Cox10 and Ndufa4 promoters in Mule-deficient hearts, suggesting that Myc directly inhibits expression of both factors (Fig. 5F).

To exclude that a global decline in mt biogenesis is the underlying mechanism for the observed reduction in mt OxPhos factors, mtDNA copy number was determined by qPCR of the mt gene cytochrome b (Cytb), normalized to levels of nuclear encoded β-actin. There was no significant change in mt copy number in *Mule*^*fl*/*fl*(*y*)^*;mcm* post-Tam as compared to untreated *Mule*^*fl*/*fl*(*y*)^*;mcm* mice (Fig. 5G). However, mt gene transcription, as a surrogate for mitochondrial capacity, was decreased in *Mule*^*fl*/*fl*(*y*)^*;mcm* hearts post-Tam and this phenomenon was absent in *DKO* mice (Fig. 5H). To confirm the protective effect of Myc ablation on mt function, mt complex (C) CI and CIV activity was assessed using immunocomplex assays. Notably, CI (Fig. 5I) and CIV (Fig. 5J) were significantly reduced in the presence of activated Myc in *Mule*^*fl*/*fl*(*y*)^*;mcm* post-Tam, while co-ablation of Myc and Mule in *DKO* mice prevented Mule-induced reduction in mt complex activity. This suggests that Myc-mediated transrepression of the Ndufa4 (CI) and Cox10 (CIV) may play a role in the negative regulation of complex I and IV activities in Mule-deficient CM (Fig. 5).

### Activation of Myc in Mule-deficient cardiomyocytes induces oxidative stress

**Figure 6:**
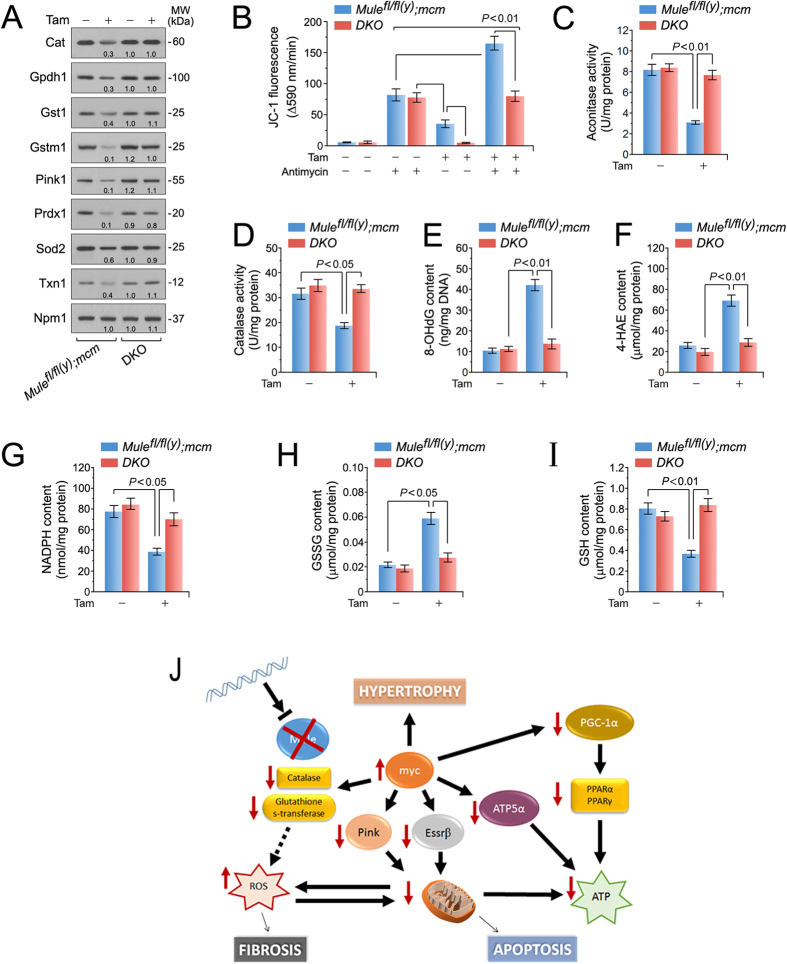
Enhanced oxidative stress in mice with cardiac-specific deletion of Mule. (**A**) Immunoblot analysis of factors involved in detoxification processes of reactive oxygen species in left ventricular extracts of *Mule*^*fl*/*fl*(*y*)^*;mcm* and *DKO* mice at 7 days post-Tam employing specific antibodies as indicated on the left. One representative result of three independent experiments is shown. (**B**) The membrane potential (ΔΨm) of mitochondria isolated from Mule mutant mice is susceptible to ROS-induced depolarization. Genetic co-ablation of Myc and Mule in *DKO* mice rescues antimycin induced decreases of ΔΨm in mitochondria derived from this double mutant strain. Isolated mitochondria, incubated with JC-1 (5 μg/mL), were treated with antimycin (50 μM). JC-1 emission at 535/595 nm was recorded at 1 reading/min for 30 min using a fluorescence spectrophotometer. The rate between two time points (Δemission at 595 nm/min) was calculated in the most linear range of decline for JC-1 fluorescence intensity. *n* = 4. (**C**,**D**) Mitochondrial aconitase (**C**) and catalase (**D**) activities were determined spectrophotometrically in ventricular samples from *Mule*^*fl*/*fl*(*y*)^*;mcm* and *DKO* strains at 7 days post-Tam. *n* = 4. (**E**) Oxidative genomic DNA damage in the hearts of *Mule*^*fl*/*fl*(*y*)^*;mcm* mice is abolished by genetic co-ablation of Myc in DKO animals. Concentrations of 8-hydroxy-2′-deoxyguanosine (8- OHdG), a biomarker for oxidative DNA damage in the indicated strains was determined by a competitive enzyme-linked ELISA employing 8-OHdG antibodies. *n* = 4. (**F**) Higher levels of 4-hydroxyalkenals (4-HAE), an indicator of ROS-dependent lipid peroxidation in ventricular extracts of *Mule*^*fl*/*fl*(*y*)^*;mcm* compared with *DKO. n* = 4. (**G**) Significantly reduced levels of NADPH, an essential cofactor for the reduction of glutathione, in in ventricul*ar extracts of *Mule*^*fl*/*fl*(*y*)^*;mcm* mice versus *DKO. n* = 4. (**H**,**I**) Decreased the reduced glutathione/oxidized glutathione (GSH/GSSG) ratios, an indicator of cardiac oxidative stress, in ventricular samples from *Mule*^*fl*/*fl*(*y*)^*;mcm* mice that was prevented by Myc co-ablation in *DKO. n* = 4. Schematic model for Mule-mediated inhibition of Myc-dependent cardiac hypertrophy. In response to genetic ablation of Mule, Myc is activated and transcriptionally inhibits canonical downstream targets Pgc-1α and Pink1 promoting the development of heart failure by induction of oxidative stress and mitochondrial dysfunction. Data are means ± s.e.m.

Pink1 and Myc play important roles in cardiac redox homeostasis^39^ and ROS generation^40^, respectively. Accordingly, we analyzed expression of antioxidant factors by immunoblotting. Protein levels of Pink1, mt Cat, Gst1, Gstm1, Prdx1, and Txn1 were markedly downregulated in *Mule*^*fl*/*fl*(*y*)^*;mcm*, but not in *DKO* post-Tam (Fig. 6A). In contrast, expression of the key antioxidant Sod2 was unaffected by Mule ablation. Since Pink1-deficient CM display reduced mt CI reductive activity and mt membrane potential^41^, we evaluated the consequence of Mule ablation on mt function. To assess integrity of transmembrane potential (ΔΨm) following acute oxidative stress, mt isolated from *Mule*^*fl*/*fl*(*y*)^*;mcm* LV samples were labeled with the fluorochrome JC-1 (2 μM) to monitor the ΔΨm, then exposed to antimycin (50 μM) to inhibit CIII and induce ROS production. As expected, we observed a ROS-dependent impairment of ΔΨm in mt from Mule-deficient cardiomyocytes compared with control samples at baseline and in the presence of antimycin (Fig. 6B), indicating enhanced ROS production. To further substantiate the hypothesis that Myc-induced Pink1-depletion leads to oxidative stress, activity of mt aconitase and mt Cat, alternate markers of oxidative stress^42^,^43^ were determined biochemically. Tam injection of *Mule*^*fl*/*fl*(*y*)^*;mcm* induced a 5.3-fold and 2-fold decrease in aconitase and Cat activities, respectively (Fig. 6C,D). This effect was blocked in *DKO* post- Tam. These data demonstrate that Mule deficiency is accompanied by elevated mt ROS and dysfunctional ROS detoxification. Moreover, this was associated with significantly higher levels of 8-hydroxy-2′-deoxyguanosine (8-OHdG), a principal oxidative lesion causing DNA mutations by base mispairing^44^, in Tam-treated *Mule*^*fl*/*fl*(*y*)^*;mcm* (Fig. 6E). Similarly, levels of cytotoxic 4-hydroxyalkenals (4-HAE), an indicator of ROS-dependent lipid peroxidation^45^, were also significantly elevated (Fig. 6F). Finally, we measured NADPH and reduced glutathione/oxidized glutathione (GSH/GSSG) ratios. NADPH, a product of the pentose phosphate pathway, is a key component in cellular anti-oxidation systems including glutathione^46^. We noted statistically significant decreases in NADPH content (Fig. 6G) and GSH/GSSG ratios (Fig. 6H,I) in Tam injected *Mule*^*fl*/*fl*(*y*)^*;mcm* hearts compared to controls. Therefore, loss of Mule promotes oxidative stress in the context of deregulated CI/CIV activity by the Myc/Pgc-1a axis, consistent with an augmentation of the effect of Pink1 on CI function (Fig. 6J).

## Discussion

In this study we report a role for Mule in differentiated cardiomyocytes where it exerts growth suppression through Myc inhibition by promoting its degradation. Mule ablation activates Myc sufficiently to induce congestive HF and early death. Notably, Mule knockout mice recapitulate decreased Mule levels in end-stage human HF. This data support an important role for Mule in maintaining cardiac homeostasis. Our work is significant in that little is currently known regarding the role of Mule in Myc-dependent metabolic changes within the heart. In addition, we provide genetic and experimental evidence that Mule transcript levels are diminished in hearts of humans diagnosed with idiopathic dilated cardiomyopathy and ischemic cardiomyopathy. We performed a comprehensive study in which multiple experimental parameters including indices of hypertrophy, mitochondrial function, antioxidant status, and mt biogenesis were systematically investigated in the Mule-deficient heart and in hearts lacking both Mule and Myc. Our findings are of interest in the field and the mouse models generated in the course of our study represent important tools for investigating various aspects of cardiac hypertrophy.

Transcriptomic analysis of mutant strains identified that elevated Myc directly inhibits transcription of Pgc-1α and Pink1, key factors regulating mitochondrial energy metabolism and ROS defense^26^,^36^,^47^. This causes decreased expression of Ndufa4 and Cox10 associated with impaired complex I, IV activity and severely impaired antioxidant levels, leaving the heart vulnerable to oxidative stress.

We found a significant downregulation of Esrrβ upon Mule ablation, while mRNA expression of Esrrα/γ remained unchanged. Little is known about the Esrrβ isoform in the heart, but our data suggests that Myc-dependent downregulation of Esrrβ may play a role in decreased expression of the mt proteins such as Ndufa4, Cox10 and Atp5a.

We have previously shown that Pink1-deficient hearts exhibit decreased complex I activity, directly leading to a drop in mt membrane potential^39^. Pink1 may also play an important role in mitophagy and cell survival^48^. Here we note that Pink1 is strongly downregulated in the absence of Mule. Activated Myc leads to ROS production which correlates strongly with the induction of DNA damage and apoptosis^40^, leading us to speculate that Myc-dependent mt dysfunction leads to ROS accumulation. Indeed, cardiac remodeling and fibrosis was heavily evident in the *Mule*^*fl*/*fl*(*y*)^*;mcm* animals, and ROS-mediated cardiac fibrosis has been well established^49^,^50^. Importantly, we observed significant downregulation of important redox regulators (Pink1, Prdx and Txn1), and antioxidants (Cat, Gpdh1, Gst1 and Gstm1) in a Myc- dependent manner, suggesting that the Mule-deficient heart has remarkably reduced ROS handling capacity. Speculatively, defective oxphos complex I caused by impaired Pink1 activity, as well as Myc-mediated transrepression of Ndufa4, and Myc-dependent decreases in oxphos complex IV and V activities, could additionally contribute to ROS production and oxidative stress after Mule ablation. Coincidently, although we failed to detect cardiomyocyte proliferation in the Myc-upregulated *Mule*^*fl*/*fl*(*y*)^*;mcm* myocardium, we noted significant recruitment of non-cardiomyocytes to intrafibrillar regions marked by excessive ECM accumulation. Taken together, Myc-mediated reduction of ROS handling leading to fibrosis are likely factors contributing to decreased contractile function following Mule ablation in the heart.

In summary, our results demonstrate that Mule plays a critical non-redundant role in maintaining cardiac homeostasis through the control of Myc protein abundance in the heart *in vivo*. In a model summarized in Fig. 6J, Myc dependent downregulation of Pgc-1α and Pink1 leads to mitochondrial dysfunction and oxidative stress regulating cardiac hypertrophy, fibrosis, and apoptosis, which impacts heart function, ultimately leading to premature mortality.

## Additional Information

**How to cite this article**: Dadson, K. *et al*. The E3 ligase Mule protects the heart against oxidative stress and mitochondrial dysfunction through Myc-dependent inactivation of Pgc-1α and Pink1. *Sci. Rep.* **7**, 41490; doi: 10.1038/srep41490 (2017).

**Publisher's note:** Springer Nature remains neutral with regard to jurisdictional claims in published maps and institutional affiliations.

## Supplementary Material

Supplementary Information
