# Supplementary material for: The E3 ligase Mule protects the heart against oxidative stress and mitochondrial dysfunction through Myc-dependent inactivation of Pgc-1α and Pink1
Source: Sci Rep. 2017 Feb 2;7:41490. doi: 10.1038/srep41490 (PMC5288653; doi:10.1038/srep41490)
Supplement: Supplementary Information [file srep41490-s1.pdf]

Supplementary Information

The E3 ligase Mule protects the heart against oxidative stress and mitochondrial dysfunction through Myc-dependent inactivation of Pgc-1α and Pink1.

Keith Dadson, Ludger Hauck, Zhenyue Hao, Daniela Grothe, Vivek Rao, Tak W. Mak, and Filio Billia

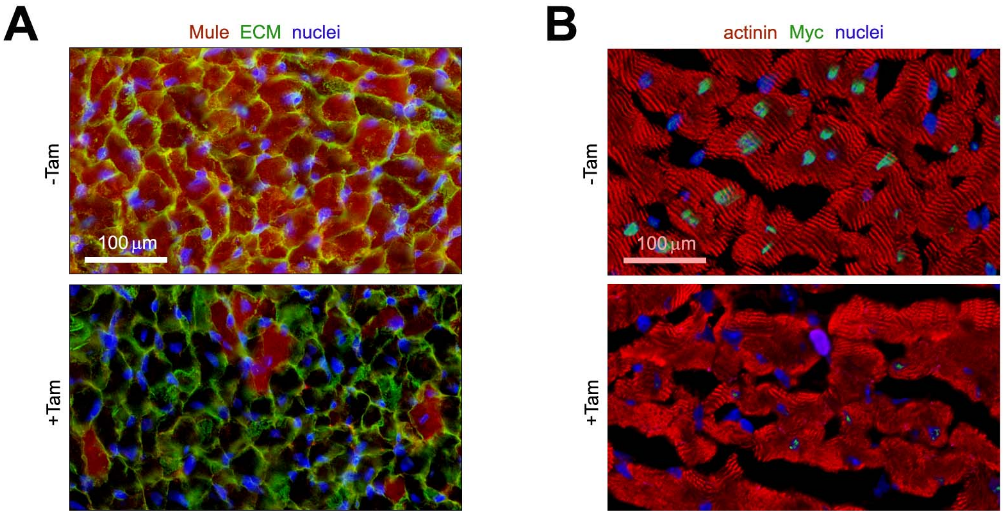

Supplementary Figure S1: Analysis of recombination efficiency.

(A, B) Confocal immunofluorescence microscopical analysis at 7d post Tam shows that four consecutive daily intraperitoneal Tam injections induced genetic ablation of **Mule and Myc** with high recombination efficiency (> 90%). Mice were 12 weeks old at the time of analysis.

(A) Administration of Tam induced marked downregulation of endogenous cytoplasmic Mule expression in left ventricular cardiomyocytes from *Mule*$^{fl/fl(y)}$*;mcm* mice. Specimen were co-stained for immunofluorescence microscopy employing Mule antibodies (red), WGA (green), and Dapi (nuclear DNA; blue).

(B) Administration of Tam significantly decreased expression levels of endogenous nuclear Myc in the heart of *Myc*$^{fl/fl}$*;mcm* mice. Left ventricular samples were treated with anti-Myc and anti-α-actinin antibodies, and co-stained with Dapi. Red, α-actinin. Green, Myc. Blue, nuclei.

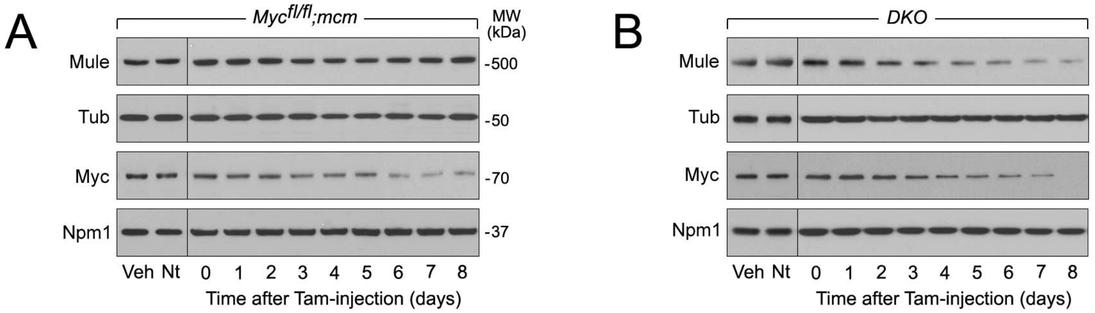

Supplementary Figure S2:

(A) Administration of Tam induced marked downregulation of endogenous nuclear Myc expression in left ventricular (LV) cardiomyocytes from *Myc*$^{fl/fl}$*;mcm* mice. Immunoblot analysis of Myc in biochemically fractionated LV extracts (60 µg total protein/lane) was done employing specific antibodies as indicated (left).

(B) Administration of Tam significantly decreased expression levels of endogenous cytoplasmic Mule and nuclear Myc in the heart of *Mule*$^{fl/fl(y)}$;*Myc*$^{fl/fl}$*;mcm* mice (DKO). LV tissue samples were analyzed by immunoblotting employing antibodies as indicated (left).

Western blots were repeated at least once with similar results.

Veh, vehicle injections. Nt, no treatment.

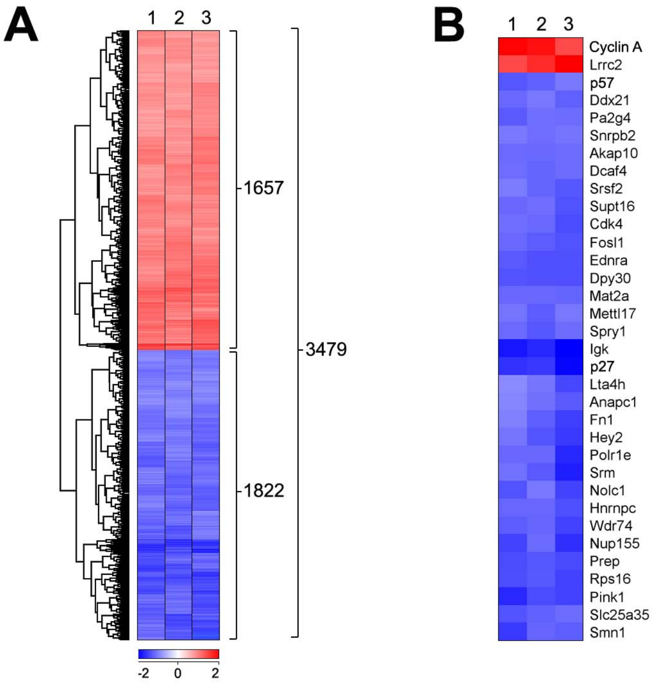

Supplementary Figure S3: Transcriptomic analysis of Mule mutant hearts.

(A) Genome-wide mRNA microarray profiling reveals that conditional genetic ablation of Mule induces profound alterations in the cardiac transcriptome indicating a most unexpected high degree of complexity associated with Mule network regulation in the heart. Heat map of unsupervised hierarchical cluster analysis at a high confidence threshold identifies 3479 transcripts (rows) out of 33561 entities that were enriched in ventricular samples of $Mule^{fl/fl(y)}$*;mcm* mice (columns) post-Tam relative to vehicle-injected controls. Mice were analyzed at 4 weeks post-Tam. Values ($\log_2$ expression) are shown by color and intensity of shading. Blue, repressed. Red, induced. $n = 3$ biological replicates. $P < 0.05$. Fold change >1.3 and < −1.3.

(B) Heat map examining the impact of genomic modifications in Mule mutant hearts (columns) on previously validated Myc target genes (rows). The majority of these factors was downregulated in $Mule^{fl/fl(y)}$*;mcm* mice post-Tam demonstrating that Myc activation is a consequence of inhibiting Mule, and that Myc is involved in the development of the mutant cardiac phenotype.

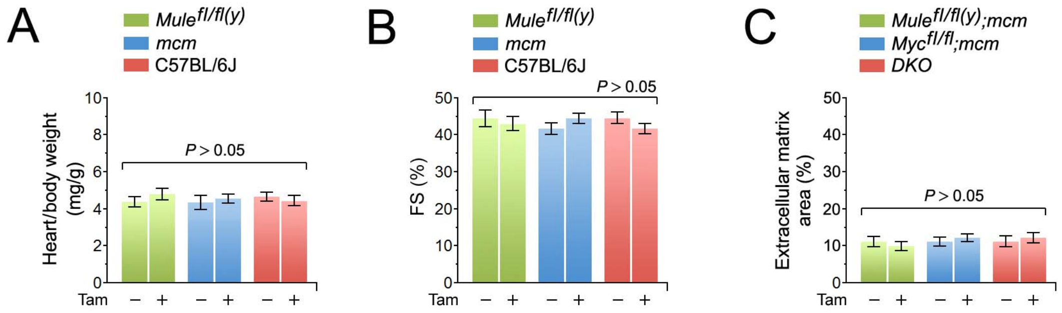

Supplementary Figure S4:

(A) Heart-weight corrected for body weight. *n*=4-6. Mice were 12 weeks old at the time of analysis.

(B) Fractional shortening (FS) determined by M-mode echocardiography. *n*=4.

(C) Quantification of extracellular matrix area by immunofluorescence microscopy employing routine wheat germ agglutinin (WGA-) stained cardiac sections. *n*=4

(A-C) Data are means ± s.e.m.

Supplementary Table S1: Primer sequences used in ChIP assays.

Gene Symbol	Forward	Reverse
**ATP5a**	ACTCGAGGACCCAGGTTTG	CCACGTGAGTAGCAGAACCA
**Bckdhb**	CTTTAAATCAGTCATTCAACTTGC	CAGACGGAAGCCATACCAAG
**Cox4i1**	TCTTGGTCTTCCGGTTGC	CACACCTCCTGCGACTAGA
**Cox10**	CACAGAAATGCTAACCTCATTCC	GGAGCTTATAAAGATCGGATGG
**Esrrb**	CCGGGAGCTTATGTCAACTG	AGAGAGCCGGTGAGTACAGG
**Got1**	ACAAAACGCCCAAGTACAGG	GTGGGGAAAGCAGAGAACAG
**Gstm1**	GGACAGTGTTCTTGGGAGGA	ACCAGAGCAGCAGACCCTAA
**Hk2**	GGAACACACGTCCCAACTCT	AGTCGGGGCCAAATAGAGAA
**Ndufa4**	GCCAGTGAAAGTCAGGAAGC	GTCACCCAGGACCTCCTACC
**Ndufa8**	TGAGCCATCTCTCCAGCTCT	GCCATTCCAGGAAGTCTAGC
**Pgc-1a**	TGTGGCGGTTTTGTTGACTA	TCCCCAGTCACATGACAAAG
**Pink1**	CAAGCAGTTTAAGTCATAGGGATTT	TGGGACAACAACAAACTTCG
**Ppara**	CCTAGGGGGCGGAGTTTC	CAGGGGACAACCAGAGGAC
**Pparg**	CAGCACCAACCACCTGTAAC	GTGCTGTGCGTCGGTGAG

Supplementary Table S2: Primer sequences used in RT-qPCR assays.

Gene Symbol	Forward	Reverse
**Acta1**	CCCAAAGCTAACCGGGAGAAG	CCAGAATCCAACACGATGCC
**Acta2**	GTCCCAGACATCAGGGAGTAA	TCGGATACTTCAGCGTCAGGA
**β-actin**	GAAATCGTGCGTGACATCAAAG	TGTAGTTTCATGGATGCCACAG
**Actn1**	GACCATTATGATTCCCAGCAGAC	CGGAAGTCCTCTTCGATGTTCTC
**Actn4**	ATGGTGGACTACCACGCAG	CAGCCTTCCGAAGATGAGAGT
**ANP**	GCTTCCAGGCCATATTGGAG	GGGGGCATGACCTCATCTT
**ATP5a1**	TCTCCATGCCTCTAACACTCG	CCAGGTCAACAGACGTGTCAG
**ATP5i2**	TGCCGAGCTGGATAATGATGC	ACCATGCTAATCCCCGAGATG
**B2m**	TTCTGGTGCTTGTCTCACTGA	CAGTATGTTCGGCTTCCCATTC
**Bckdhb**	AGCTATTGCGGAAATCCAGTTT	ACAGTTGAAAAGATCACCTGAGC
**Bckdk**	ACATCAGCCACCGATACACAC	GAGGCGAACTGAGGGCTTC
**BNP**	GAGGTCACTCCTATCCTCTGG	GCCATTTCCTCCGACTTTTCTC
**Cat**	AGCGACCAGATGAAGCAGTG	TCCGCTCTCTGTCAAAGTGTG
**Cox4i1**	ATTGGCAAGAGAGCCATTTCTAC	CACGCCGATCAGCGTAAGT
**Cox10**	AGAAGAGCTATACAGGGATTGCC	CTGTGTGACATACATGCGCTT
**Cytb**	TTCTGAGGTGCCACAGTTATT	GAAGGAAAGGTATTAGGGCTAAA
**Got1**	GCGCCTCCATCAGTCTTTG	ATTCATCTGTGCGGTACGCTC
**Gpi1**	TCAAGCTGCGCGAACTTTTTG	GGTTCTTGGAGTAGTCCACCAG
**Gpt2**	AACCATTCACTGAGGTAATCCGA	GGGCTGTTTAGTAGGTTTGGGTA
**Gsta3**	AAGAATGGAGCCTATCCGGTG	CCATCACTTCGTAACCTTGCC
**Gstm1**	ATACTGGGATACTGGAACGTCC	AGTCAGGGTTGTAACAGAGCAT
**Gstp1**	ATGCCACCATACACCATTGTC	GGGAGCTGCCCATACAGAC
**Gstp2**	CATTACAGGGCAGCAGGAGT	CATATGGCTCCCTCTTGTCC
**Hk1**	AGGGCGCATTACTCCAGAG	CCCTGTGGGTGTCTTGTGTG
**Hk2**	TGATCGCCTGCTTATTCACGG	AACCGCCTAGAAATCTCCAGA

Supplementary Table S2: Primer sequences used in RT-qPCR assays (continued).

Gene Symbol	Forward	Reverse
**α-MHC**	GCCCAGTACCTCCGAAAGTC	GCCTTAACATACTCCTCCTTGTC
**α-MHC**	GCCCAGTACCTCCGAAAGTC	GCCTTAACATACTCCTCCTTGTC
**β-MHC**	ACTGTCAACACTAAGAGGGTCA	TTGGATGATTTGATCTTCCAGGG
**Ndufa4**	TCCCAGCTTGATTCCTCTCTT	GGGTTGTTCTTTCTGTCCCAG
**Ndufa8**	GGAGCTGCCAACTCTGGAAG	CCAGCGGCACAGCATAAAC
**Ndufv1**	TTTCTCGGCGGGTTGGTTC	GGTTGGTAAAGATCCGGTCTTC
**Pgc-1α**	TATGGAGTGACATAGAGTGTGCT	CCACTTCAATCCACCCAGAAAG
**Pgc-1β**	TCCTGTAAAAGCCCGGAGTAT	GCTCTGGTAGGGGCAGTGA
**Pink1**	TTCTTCCGCCAGTCGGTAG	CTGCTTCTCCTCGATCAGCC
**Pparα**	AGAGCCCCATCTGTCCTCTC	ACTGGTAGTCTGCAAAACCAAA
**Pparγ**	TCGCTGATGCACTGCCTATG	GAGAGGTCCACAGAGCTGATT
**Tgm2**	GACAATGTGGAGGAGGGATCT	CTCTAGGCTGAGACGGTACAG
**Tnnc1**	GCGGTAGAACAGTTGACAGAG	CCAGCTCCTTGGTGCTGAT
**Tnnt1**	CCTGTGGTGCCTCCTTTGATT	TGCGGTCTTTTAGTGCAATGAG

Supplementary Table S3: Antibodies employed for ChIP, IF, IP and WB.

Gene name	Catalog No.	Vendor	Application	Dilution Factor
α-actinin, sarcomeric	A7811	Sigma	IF	50
Atp5a	ab14748	Abcam	WB	1000
Atp5b	ab14730	Abcam	WB	1000
BrdU	MCA2060	AbD Serotec	IF	50
Cat	14097	Cell Signaling	WB	1000
Complex I	ab109798	Abcam	IP	5 μg
Complex IV	ab109863	Abcam	IP	5 μg
Cox4i1	ab16056	Abcam	WB	1000
Cox10	ab84053	Abcam	WB	1000
CytC	ab13575	Abcam	WB	1000
Gpdh1	ab87230	Abcam	WB	1000
Gst1	ab53940	Abcam	WB	1000
Gstm1	ab178684	Abcam	WB	1000
Pink1	3929-100	BioVision	WB	1000
Prdx1	8499	Cell Signaling	WB	1000
Sod2	13194	Cell Signaling	WB	1000
Mef2a	ab32866	Abcam	IF	50
Mule	Ab70161	Abcam	IF WB	50/1000
Myc	sc-764	Santa Cruz	IF IP<br>WB ChIP	50 - 7.5μg<br>1000 - 7.5 μg
Ndufa4	ab129752	Abcam	WB	1000
Ndufa8	BS3336	BioWorld	WB	1000
normal rabbit IgG	2729	Cell Signaling	CHIP	7.5 μg
Npm1	B0556	Sigma	WB	1000
Opa1	ab42364	Abcam	WB	1000
Pgc-1α	3934-100	BioVision	WB	1000
Pparα	PAB11321	Abnova	WB	1000
Pparγ	PAB11321	Abnova	WB	1000
Sdhb	ab14714	Abcam	WB	1000
ubiquitin	13-1600	Thermo Fisher	WB	7.5 μg
tubulin	2146	Cell Signaling	WB	1000

ChIP, chromatin immunoprecipitation. IF, immunofluorescence microscopy. IP, immunoprecipitation. WB, Western blot.

Supplementary Table S4: Patient characteristics of human LV samples as in Fig. 1A.

Etiology of HF syndrome	Age (years)	Sex
**Familial DCM**	47	Female
**Familial DCM**	50	Female
**Idiopathic DCM**	51	Female
**Idiopathic DCM**	47	Male
**Idiopathic DCM**	24	Female
**Idiopathic DCM**	55	Male
**Idiopathic DCM**	48	Male
**Idiopathic DCM**	59	Male
**Idiopathic DCM**	60	Female
**Idiopathic DCM**	71	Female
**Idiopathic DCM**	64	Male
**Idiopathic DCM**	61	Male
**Idiopathic DCM**	44	Female
**Idiopathic DCM**	24	Female
**Idiopathic DCM**	26	Male
**Idiopathic DCM**	58	Male
**Idiopathic DCM**	56	Female
**Idiopathic DCM**	46	Male
**Congenital CM**	16	Female
**Congenital CM**	31	Male
**Congenital CM**	57	Female
**Ischemic CM**	46	Male
**Ischemic CM**	45	Male
**Ischemic CM**	61	Male
**Ischemic CM**	69	Male
**Ischemic CM**	58	Male
**Ischemic CM**	53	Male
**Ischemic CM**	51	Male
**Ischemic CM**	67	Male

Informed consent was obtained from patients with end-stage HF who were to have a left ventricular assist device inserted as either a bridge to a heart transplant or as destination therapy to end of life. Samples were obtained from the LV apex and frozen in liquid nitrogen at the time of procurement.

Supplementary Table S4: Patient characteristics of human LV samples as in Fig. 1A (continued).

Etiology of HF syndrome	Age (years)	Sex
**Ischemic CM**	44	Male
**Ischemic CM**	59	Male
**Ischemic CM**	62	Male
**Ischemic CM**	66	Male
**Ischemic CM**	58	Male
**Myocarditis**	57	Male
**Myocarditis**	50	Male
**Myocarditis**	41	Male
**Chemotherapy-induced CM**	55	Male
**Chemotherapy-induced CM**	51	Female
**Chemotherapy-induced CM**	36	Male
**Chemotherapy-induced CM**	62	Female
**Control**	24	Male
**Control**	21	Male
**Control**	27	Male
**Control**	26	Male
**Control**	21	Male
**Control**	24	Male
**Control**	27	Male
**Control**	24	Male

Informed consent was obtained from patients with end-stage HF who were to have a left ventricular assist device inserted as either a bridge to a heart transplant or as destination therapy to end of life. Samples were obtained from the LV apex and frozen in liquid nitrogen at the time of procurement.
